# Prevalence, Associated Factors, and Survival Outcomes of Small-Cell Neuroendocrine Carcinoma of the Gynecologic Tract: A Large Population-Based Analysis

**DOI:** 10.3389/fmed.2022.836910

**Published:** 2022-04-11

**Authors:** Li Pang, Shizhuo Wang

**Affiliations:** Department of Obstetrics and Gynecology, Shengjing Hospital of China Medical University, Shenyang, China

**Keywords:** small cell carcinoma, neuroendocrine carcinoma, prevalence, prognosis, ovarian epithelial carcinoma, endometrial neoplasms, cervical carcinoma

## Abstract

Small-cell carcinomas are highly malignant tumors with neuroendocrine function and which often occur in the lungs. Primary small-cell neuroendocrine carcinomas of the gynecologic tract are extremely rare. This study aimed to evaluate the prevalence of independent predictors related to the prognosis and overall survival of patients with small-cell neuroendocrine carcinomas of the gynecologic tract. Patients with gynecologic small-cell neuroendocrine carcinomas diagnosed between 1973 and 2015 were identified from the Surveillance, Epidemiology, and End Results database. Univariate and multivariate Cox risk regression analyses were performed to determine the independent predictors of overall survival. Overall survival was calculated using the Kaplan–Meier method, and the log-rank test was used for comparison. We included 5,15,393 eligible carcinomas in the present study; the prevalence of gynecologic small-cell neuroendocrine carcinomas was 0.121% (*N* = 622). Multivariate analysis indicated that advanced age, stage III and IV cancer, and no chemotherapy treatment may be predictors of poor small-cell neuroendocrine cervical carcinoma prognosis. Stage III and IV cancer and lack of surgery, radiotherapy, or chemotherapy were identified as potential predictors of poor prognosis in patients with ovarian small-cell neuroendocrine carcinoma. Kaplan–Meier analysis suggested that the median survival was 19, 11, and 12 months for cervical, ovarian, and endometrial small-cell neuroendocrine carcinomas, respectively. The 1-, 3-, and 5-year overall survival rates were as follows: 58.8, 31.4, and 26.1%, respectively, for small-cell neuroendocrine cervical carcinoma; 46.3, 23.5, and 22.0%, respectively, for ovarian small-cell neuroendocrine carcinoma; and 49.4, 29.4, and 25.9%, respectively, for endometrial small-cell neuroendocrine carcinoma. Our findings indicate that comprehensive and individualized treatment of small-cell neuroendocrine carcinomas of the gynecologic tract may prolong patient survival, although further studies are required.

## Introduction

Small-cell carcinomas are highly malignant tumors with neuroendocrine function, often occurring in the lungs. Primary small-cell neuroendocrine carcinomas of the gynecologic tract are extremely rare; they most often occur in the cervix, followed by the ovary and endometrium (1).

While case studies and studies of single gynecological neuroendocrine cancers exist (2, 3), no large-scale studies have investigated treatment strategies and patient prognosis across different areas of the gynecologic tract. Additionally, there is currently no conventional guideline, unified treatment plan, or method for prognostic evaluation for gynecologic small-cell neuroendocrine carcinoma. This study aimed to investigate the prevalence, risk factors, and survival outcomes of gynecologic small-cell neuroendocrine carcinoma using the National Cancer Institute’s Surveillance, Epidemiology, and End Results (SEER) dataset.

## Materials and Methods

### Data Source and Patient Selections

The patients and related data were recruited and obtained from the SEER database, respectively. We enrolled patients with primary gynecologic small-cell neuroendocrine carcinoma initially diagnosed between 1973 and 2015. The site International Classification of Diseases for Oncology 3 code was 8041/3 (small-cell carcinoma), while the World Health Organization 2008 designations were restricted to cervical, ovarian, and uterine corpus. The exclusion criteria were as follows: age <18 years, diagnosis of carcinoma *in situ*, benign or borderline tumors, diagnosis at autopsy or *via* death certificate, presence of a non-primary gynecologic small-cell neuroendocrine carcinoma, missing/unknown cause of death, and miscellaneous malignant cancer. SEER*Stat 8.3.8 software^1^ was used to generate case listings. The deidentified data in the SEER database are publicly available; thus, their use is exempt from review by the Shengjing Hospital Affiliated to China Medical University Institutional Review Board.

### Clinical and Demographic Characteristics

We analyzed the patients’ demographic data, including age at diagnosis (<45, 45–64, 65–84, ≥85 years), race (white, black, other, and unknown), marital status (single/unmarried, married, divorced/separated, widowed, and unknown), insurance status (insured, any Medicaid, uninsured, and unknown), year of diagnosis (1973–2015), tumor stage and grade (IA-IVB or unknown; well-differentiated, moderately differentiated, poorly differentiated, undifferentiated, or unknown), lymph node status (negative, positive, not examined, and unknown), number of lymph node metastases (none, 1–3, ≥4, and unknown), bone metastasis (yes, no), brain metastasis (yes, no), liver metastasis (yes, no), and lung metastasis (yes, no). Treatment patterns included surgery (yes, no), chemotherapy (yes, no), and radiotherapy (yes, no).

### Statistical Analysis

The patients’ clinical and demographic characteristics were compared using chi-square tests or Fisher’s exact test. Categorical data are presented as numbers and percentages (N,%). Quantitative data are presented as the mean ± standard deviation. Univariate and multivariate Cox risk regression analyses were performed to determine independent predictors of overall survival. Overall survival was calculated using the Kaplan–Meier method, and the log-rank test was used for comparison. All data were analyzed using SPSS software (version 23.0; SPSS, Chicago, IL, United States). Kaplan–Meier survival curves were drawn using GraphPad Prism (GraphPad Software, San Diego, CA, United States), and *P*-values <0.05 were considered significant.

In accordance with the journal’s guidelines, we will provide our data for the reproducibility of this study in other centers upon request.

## Results

The prevalence of cervical small-cell neuroendocrine carcinoma was higher among married, divorced/separated, and widowed patients than among single/unmarried patients (*P* = 0.001) and among uninsured patients and those with any form of Medicaid than among insured patients (*P* < 0.001). Stages II–IV were associated with a higher prevalence than stage I (*P* < 0.001).

Additionally, diagnosis between 2004 and 2015 was associated with a higher prevalence of cervical small-cell neuroendocrine carcinoma than diagnosis before 2004 (*P* < 0.001). The prevalence was also higher for moderately differentiated, poorly differentiated, and undifferentiated tumors than for well-differentiated tumors (*P* < 0.001). Positive lymph nodes or non-examined lymph nodes were associated with a higher prevalence of cervical small-cell neuroendocrine carcinoma than negative lymph nodes (*P* < 0.001). Prevalence was also higher among patients with bone, brain, liver, and lung metastases than those without (Table 1).

**TABLE 1:** Demographic and clinical characteristics of patients with small-cell neuroendocrine carcinoma of the gynecologic tract (SCNCGT) and carcinoma of the gynecologic tract (CGT).

Patient	SCNCC	CC		SCNCO	OC		SCNCE	EC	
characteristics	N(%)	N (%)	*P-value*	N (%)	N (%)	*P-value*	N (%)	N (%)	*P-value*
ALL	299(0.32)	94179(99.68)		217(0.15)	145595(99.85)		106(0.04)	275619(99.96)	
**Age at diagnosis (years)**			0.41			**<0.001**			0.16
<45	137(45.8)	37952(40.3)		135(62.2)	18933(13.0)		7(6.6)	19742(7.2)	
45–64	116(38.8)	36171(38.4)		36(16.6)	59788(41.1)		42(39.6)	133542(48.5)	
65–84	43(14.4)	17524(18.6)		41(18.9)	56839(39.0)		49(46.2)	110212(40.0)	
≥85	3(1.0)	2532(2.7)		5(2.3)	10035(6.9)		8(7.6)	12123(4.3)	
**Race**			0.094			0.685			0.169
White	211(70.6)	70994(75.4)		184(84.8)	124025(85.2)		84(79.2)	232111(84.2)	
Black	46(15.4)	13556(14.4)		14(6.5)	10856(7.5)		14(13.2)	22617(8.2)	
Other	40(13.4)	8811(9.4)		18(8.3)	10294(7.1)		8(7.6)	19395(7.0)	
Unknown	2(0.6)	818(0.8)		1(0.4)	420(0.2)		0(0.00)	1496(0.6)	
**Marital status**			**<0.001**			**<0.001**			**0.007**
Single/unmarried	95(31.8)	20585(21.9)		88(40.5)	23716(16.3)		17(16.0)	45214(16.4)	
Married	114(38.1)	41797(44.4)		90(41.5)	70767(48.6)		39(36.8)	141684(51.4)	
Divorced/separated	45(15.1)	13663(14.5)		18(8.3)	14925(10.3)		14(13.2)	24359(8.8)	
Widowed	32(10.7)	12564(13.3)		14(6.5)	30838(21.2)		30(28.3)	51591(18.8)	
Unknown	13(4.3)	5570(5.9)		7(3.2)	5349(3.6)		6(5.7)	12771(4.6)	
**Insurance status**			**<0.001**			**0.006**			**0.017**
Insured	148(49.5)	18486(19.6)		72(33.2)	41788(28.7)		32(30.2)	95130(34.5)	
Any medicaid	62(20.7)	8802(9.3)		24(11.1)	6623(4.5)		11(10.4)	12520(4.5)	
Uninsured	22(7.4)	2124(2.3)		4(1.8)	1999(1.4)		2(1.8)	3766(1.4)	
Unknown	67(22.4)	64767(68.8)		117(53.9)	95185(65.4)		60(56.6)	164203(59.6)	
**Stage**			**<0.001**			0.856			**<0.001**
I	61(20.4)	18441(19.6)		31(14.3)	14967(10.3)		7(6.6)	90296(32.8)	
II	16(5.4)	5298(5.6)		9(4.1)	5295(3.6)		2(1.8)	9606(3.5)	
III	90(30.1)	8129(8.6)		41(18.9)	22656(15.6)		20(18.8)	17635(6.4)	
IV	126(42.1)	8818(9.4)		53(24.4)	25284(17.4)		30(28.3)	16770(6.1)	
Unknown	6(2.0)	53493(56.8)		83(38.3)	77393(53.1)		47(44.3)	141312(51.2)	
**Year of diagnosis**			**<0.001**			**<0.001**			0.622
<2004	0(0.00)	52949(56.2)		81(37.3)	75539(51.9)		47(44.3)	130000(47.1)	
2004–2006	63(21.1)	10339(11.0)		29(13.4)	17170(11.8)		11(10.4)	30463(11.1)	
2007–2009	76(25.4)	10548(11.2)		42(19.4)	17708(12.2)		18(17.0)	34533(12.5)	
2010–2012	68(22.7)	10162(10.8)		28(12.9)	17522(12.0)		12(11.3)	38478(14.0)	
2013–2015	92(30.8)	10181(10.8)		37(17.0)	17656(12.1)		18(17.0)	42145(15.3)	
**Grade**			**<0.001**			**<0.001**			**<0.001**
Well differentiated	1(0.3)	7816(8.3)		0(0.00)	10321(7.1)		3(2.8)	93778(34.0)	
Moderately differentiated	1(0.3)	23526(25.0)		1(0.5)	18686(12.8)		1(1.0)	68926(25.0)	
Poorly differentiated	110(36.8)	24415(25.9)		47(21.7)	41580(28.5)		36(34.0)	45577(16.5)	
Undifferentiated	63(21.1)	2361(2.5)		73(33.6)	15085(10.4)		40(37.7)	14291(5.2)	
Unknown	124(41.5)	36061(38.3)		96(44.2)	59923(41.2)		26(24.5)	53047(19.2)	
**Lymph nodes status**			**<0.001**			**0.049**			**<0.001**
Negative	48(16.1)	20707(22.0)		49(22.6)	31304(21.5)		16(15.1)	99960(36.3)	
Positive	49(16.4)	6281(6.7)		37(17.1)	15053(10.3)		25(23.6)	16289(5.9)	

*SCNCC, small-cell neuroendocrine carcinoma of the cervix; SCNCO, small-cell neuroendocrine carcinoma of the ovary; SCNCE, small-cell neuroendocrine carcinoma of the endometrium; SNNCC, small-cell neuroendocrine carcinoma of the cervix; OC, carcinoma of the ovary; EC, carcinoma of the endometrium; NA, Not available. All factors with unknown data were removed in logistic regression. Bold value means p < 0.05.*

Treatment patterns differed between the cervical small-cell neuroendocrine carcinoma and cervical cancer groups. Patients with cervical small-cell neuroendocrine carcinoma were more likely to receive adjuvant chemotherapy (77.9% vs. 29.5%; *P* < 0.001) and adjuvant radiotherapy (28.0% vs. 47.6%; *P* < 0.001) than those with cervical cancer. Additionally, patients with cervical small-cell neuroendocrine carcinoma were less likely to receive adjuvant surgery than those with cervical cancer (40.5% vs. 50.7%; *P* < 0.001; Table 1).

The prevalence of ovarian small-cell neuroendocrine carcinoma was higher in the ≥85-year age group than in the 45–64-year age group (*P* < 0.001) Furthermore, diagnosis between 2004 and 2015 was associated with a higher prevalence of ovarian small-cell neuroendocrine carcinoma than diagnosis prior to 2004 (*P* < 0.001). The prevalence of ovarian small-cell neuroendocrine carcinoma was also higher for moderately differentiated, poorly differentiated, and undifferentiated tumors than for well-differentiated tumors (*P* < 0.001). Positive or non-examined lymph nodes were associated with a higher prevalence of ovarian small-cell neuroendocrine carcinoma than negative lymph nodes (*P* = 0.049). The prevalence was also higher among patients with bone, brain, and liver metastases than those without (Table 1).

Treatment patterns differed between the ovarian small-cell neuroendocrine carcinoma and ovarian cancer groups. Patients with ovarian small-cell neuroendocrine carcinoma were more likely to receive adjuvant chemotherapy (73.7% vs. 59.6%) (*P* < 0.001) and adjuvant radiotherapy (7.40% vs. 3.1%; *P* < 0.001) than those with ovarian cancer. Additionally, the rate of adjuvant surgery was not significantly different between these groups (80.6% vs. 75.4%; *P* = 0.166) (Table 1).

The prevalence of endometrial small-cell neuroendocrine carcinoma was higher among married, divorced/separated, and widowed patients than among single/unmarried patients (*P* = 0.007). Stages II–IV were also associated with a higher prevalence than stage I (*P* < 0.001). Prevalence was also higher for moderately differentiated, poorly differentiated, and undifferentiated tumors than for well-differentiated tumors (*P* < 0.001). Positive or non-examined lymph nodes were associated with a higher prevalence of endometrial small-cell neuroendocrine carcinoma than negative lymph nodes (*P* < 0.001). The prevalence of endometrial small-cell neuroendocrine carcinoma was also higher among patients with brain, liver, and lung metastases than those without (Table 1).

Treatment patterns differed between the endometrial small-cell neuroendocrine carcinoma and endometrial cancer groups. Patients with endometrial small-cell neuroendocrine carcinoma were more likely to receive adjuvant chemotherapy than those with endometrial cancer (57.5% vs. 13.1%) (*P* < 0.001). Additionally, patients with endometrial small-cell neuroendocrine carcinoma were less likely to receive adjuvant radiotherapy (15.1% vs. 25.6%; *P* < 0.001) and adjuvant surgery (60.4% vs. 89.5%; *P* < 0.001) than those with endometrial cancer (Table 1).

In the univariate Cox regression analysis, age, marital status, stage, lymph node status, lymph node metastasis, bone metastasis, liver metastasis, lung metastasis, surgery, chemotherapy, and radiotherapy were positively associated with risk of cervical small-cell neuroendocrine carcinoma. Age, marital status, stage, lymph node status, lymph node metastasis, liver metastasis, surgery, chemotherapy, and radiotherapy were positively associated with risk of ovarian small-cell neuroendocrine carcinoma. Furthermore, age, stage, lymph node status, lymph node metastasis, liver metastasis, surgery, chemotherapy, and radiotherapy were positively associated with risk of endometrial small-cell neuroendocrine carcinoma (Table 2).

**TABLE 2:** Univariable Cox regression for analyzing the associated factors for developing small-cell neuroendocrine carcinoma of the gynecologic tract (SCNCGT).

Subject characteristics	SCNCC		SCNCO		SCNCE	
	HR (95%CI)	*P*-value	HR (95%CI)	*P*-value	HR (95%CI)	*P*-value
**Age at diagnosis (years)**		**<0.001**		**<0.001**		**0.004**
<45	Reference		Reference		Reference	
45–64	2.098(1.502–2.928)	**<0.001**	0.251(0.101–0.623)	0.003	0.101(0.91–1.023)	0.007
65–84	3.486(2.308–5.265)	**<0.001**	0.279(0.105–0.740)	0.010	0.149(0.109–1.197)	0.073
≥85	3.073(0.963–9.807)	0.058	0.647(0.253–1.656)	0.364	0.329(0.147–0.739)	0.007
**Race**		0.152		0.875		0.213
White	Reference		Reference		Reference	
Black	1.545(1.050–2.275)	0.027	1.061(0.574–1.960)	0.850	1.684(0.921–3.079)	0.091
Other	1.254(0.826–1.903)	0.288	0.874(0.495–1.544)	0.643	0.897(0.387–2.020)	0.799
Unknown	NA	NA	NA	NA		NA
**Marital status**		**0.014**		**<0.001**		0.115
Single/unmarried	Reference		Reference		Reference	
Married	0.997(0.694–1.433)	0.988	1.240(0.882–1.745)	0.216	1.079(0.522–2.232)	0.836
Divorced/separated	1.293(0.831–2.012)	0.255	1.255(0.702–2.244)	0.443	0.913(0.360–2.318)	0.849
Widowed	2.025(1.259–3.259)	0.004	5.652(3.085–10.354)	0.000	1.853(0.895–3.834)	0.096
Unknown	NA	NA				
**Insurance status**		0.441		0.519		0.334
Insured	Reference		Reference		Reference	
Any medicaid	1.183(0.803–1.743)	0.394	1.341(0.758–2.377)	0.312	1.267(0.512–3.131)	0.609
Uninsured	1.384(0.784–2.443)	0.263	0.736(0.179–3.032)	0.671	1.455(0.883–2.398)	0.141
Unknown	NA	NA				
**Stage**		**<0.001**		**<0.001**		**0.001**
I	Reference		Reference		Reference	
II	1.322(0.531–3.295)	0.549	3.220(1.225–8.462)	0.018	1.119(0.428–1.508)	0.981
III	2.428(1.455–4.054)	0.001	2.751(1.460–5.185)	0.002	2.442(0.523–11.398)	0.256
IV	4.900(3.017–7.959)	**<0.001**	4.204(2.272–7.778)	**<0.001**	11.294(2.488–51.270)	0.002
Unknown	NA	NA	NA	NA	NA	NA
**Year of diagnosis**		0.726		0.794		0.082
<2004	NA	NA	Reference		Reference	
2004–2006	Reference		1.019(0.640–1.622)	0.936	0.900(0.434–1.865)	0.777
2007–2009	0.971(0.658–1.433)	0.883	0.975(0.646–1.471)	0.903	0.420(0.202–0.871)	0.020
2010–2012	0.844(0.560–1.272)	0.418	0.955(0.585–1.559)	0.853	0.700(0.326–1.502)	0.360
2013–2015	0.814(0.525–1.262)	0.359	0.714(0.419–1.217)	0.216	1.426(0.734–2.773)	0.295
**Grade**		0.325		0.269		0.223
Well differentiated	Reference		–	–	–	–
Moderately differentiated	0.389(0.24–6.307)	0.506	Reference		0.391(0.039–3.891)	0.423
Poorly differentiated	0.231(0.031–1.699)	0.150	1.183(0.711–1.968)	0.519	0.425(0.057–3.189)	0.405
Undifferentiated	0.200(0.027–1.488)	0.116	0.753(0.459–1.237)	0.263	0.507(0.068–3.781)	0.508
Unknown	NA	NA	NA	NA	NA	NA
**Lymph nodes status**		<**0.001**		<**0.001**		**0.003**
Negative	Reference		Reference		Reference	
Positive	1.931(1.040–3.583)	0.037	2.237(1.307–3.826)	0.003	2.562(1.056–6.218)	0.038
No examined	3.348(1.989–5.637)	<**0.001**	2.781(1.796–4.307)	**<0.001**	3.914(1.738–8.815)	0.001
Unknown	NA	NA	NA	NA	NA	NA
**LN metastasis numbers**		***p<*0.001**		**<0.001**		**0.002**
No	Reference		Reference		Reference	
1–3	1.241(0.607–2.537)	0.554	0.447(0.228–0.878)	0.019	0.508(0.175–1.476)	0.214
≥4	0.390(0.268–0.570)	**<0.001**	0.367(0.226–0.595)	**<0.001**	0.240(0.109–0.531)	**<0.001**
Unknown	NA	NA	NA	NA	NA	NA
**Bone metastasis**		**<0.001**		0.491		0.109
Yes	Reference		Reference		Reference	
No	0.369(0.198–0.688)		0.606(0.143–2.559)		0.305(0.065–1.421)	
unknown	NA	NA	NA	NA	NA	NA
**Brain metastasis**		0.123		0.272		
Yes	Reference		Reference		–	–
No	0.346(0.084–1.421)	0.141	0.047(0.143–1.827)		–	–
Unknown	NA	NA	NA	NA	–	–
**Liver metastasis**		**<0.001**		**<0.001**		**0.011**
Yes	Reference		Reference		Reference	
No	3.341(1.944–5.744)		0.249(0.116–0.535)		0.269(0.091–0.796)	
Unknown	NA	NA	NA	NA	NA	NA
**Lung metastasis**		**0.006**		**0.007**		0.129
Yes	Reference		Reference		Reference	
No	0.400(0205–0.783.)		0.168(0.038–0.743)		0.444(0.156–1.267)	
Unknown	NA	NA	NA	NA	NA	NA
**Surgery performed**		***p<*0.001**		**<0.001**		**<0.001**
Surgery	Reference		Reference		Reference	
No surgery	2.308(1.675–3.179)		3.131(2.157–4.546)		0.268(0.166–0.435)	
Unknown	NA	NA	NA	NA	NA	NA
**Chemotherapy**		**<0.001**		**<0.001**		0.005
Yes	Reference		Reference		Reference	
No	2.170(1.550–3.037)		2.262(1.619–3.160)		0.531(0.38–0.835)	
**Radiotherapy**		**0.001**		**0.015**		**0.001**
Yes	Reference		Reference		Reference	
No	0.534(0.369–0.773)		2.562(1.210–5.467)		0.209(0.090–0.488)	

*SCNCC, small-cell neuroendocrine carcinoma of the cervix; SCNCO, small-cell neuroendocrine carcinoma of the ovary; SCNCE, small-cell neuroendocrine carcinoma of the endometrium all factors with Unknown data were removed in logistic regression model. Bold value means p < 0.05.*

In the multivariable Cox regression analysis, risk of death significantly increased in patients with cervical small-cell neuroendocrine carcinoma diagnosed at 45–64 or 65–84 years of age (hazard ratio [HR]: 1.523; 95% confidence interval [CI]: 1.034–2.242 and HR: 2.581; 95% CI: 1.607–4.146, respectively). All patients with cervical small-cell neuroendocrine carcinoma who presented with stages III–IV had a higher death risk than those with other stages; the HRs were 1.993 (95% CI: 1.033–3.847) and 3.020 (95% CI: 1.602–5.693), respectively. Patients with cervical small-cell neuroendocrine carcinoma not treated with adjuvant chemotherapy also had a higher death risk (HR: 2.784, 95% CI: 1.902–4.075) than those who received adjuvant chemotherapy. The death risk was significantly higher in patients with ovarian small-cell neuroendocrine carcinoma diagnosed at stages III–IV than those diagnosed at other stages (III: HR: 3.512, 95% CI: 1.617–7.629; IV: HR: 3.836, 95% CI: 1.744–8.437). Patients with ovarian small-cell neuroendocrine carcinoma who were not treated with adjuvant surgery, chemotherapy, and radiotherapy also had a higher death risk than patients who received adjuvant surgery, chemotherapy, and radiotherapy, with HRs of 2.127 (95% CI: 1.103–4.101), 2.023 (95% CI: 1.152–3.551), and 5.988 (95% CI: 1.350–26.560), respectively. Moreover, patients with endometrial small-cell neuroendocrine carcinoma not treated with adjuvant surgery and chemotherapy also had a higher death risk than patients who received adjuvant surgery and chemotherapy, with HRs of 4.842 (95% CI: 1.552–15.108) and 2.102 (95% CI: 0.964–4.584), respectively (Table 3).

**TABLE 3:** Multivariable Cox regression for analyzing the prognosis factors for small-cell neuroendocrine carcinoma of the gynecologic tract (SCNCGT).

Subject characteristics	SCNCC		SCNCO		SCNCE	
	HR (95%CI)	*P*-value	HR (95%CI)	*P*-value	HR (95%CI)	*P*-value
**Age at diagnosis (years)**		**0.001**		0.557		
<45	Reference		Reference			
45–64	1.523(1.034–2.242)	0.033	0.825(0.302–2.250)	0.707		
65–84	2.581(1.607–4.146)	<0.001	0.526(0.172–1.606)	0.260		
≥85	2.343(0.673–8.162)	0.181	0.808(0.288–2.269)	0.686		
**Stage**		<**0.001**		**0.005**		
I	Reference		Reference			–
II	0.731(0.272–1.962)	0.534	1.816(0.590–5.586)	0.298	Reference	0.981
III	1.993(1.033–3.847)	0.040	3.512(1.617–7.629)	0.002	1.372(0.258–7.289)	0.710
IV	3.020(1.602–5.693)	<**0.001**	3.836(1.744–8.437)	0.001	5.120(0.954–27.472)	0.057
Unknown	NA	NA	NA	NA	NA	NA
**Lymph nodes status**		0.223		0.157		0.813
Negative	Reference		Reference		Reference	
Positive	3.513(0.665–18.571)	0.139	0.424(0.114–1.578)	0.201	1.044(0.255–4.277)	0.953
No examined	1.621(0.82–6.870)	0.512	0.355(0.123–1.025)	0.056	0.711(0.151–3.343)	0.666
Unknown	NA	NA	NA	NA	NA	NA
**LN metastasis numbers**		0.468		0.509		0.737
No	Reference		Reference		Reference	
1–3	1.138(0.477–2.715)	0.771	0.537(0.165–1.750)	0.302	1.679(0.416–6.776)	0.466
≥4	2.521(0.573–11.096)	0.221	0.780(0.329–1.848)	0.572	1.064(5.000–3.875)	0.924
Unknown	NA	NA	NA	NA	NA	NA
**Surgery performed**		0.550		**0.024**		**0.007**
Surgery	Reference		Reference		Reference	
No surgery	1.189(0.674–2.097)		2.127(1.103–4.101)		4.842(1.552–15.108)	
Unknown	NA	NA	NA	NA	NA	NA
**Chemotherapy**		<**0.001**		**0.014**		**0.042**
Yes	Reference		Reference		Reference	
No	2.784(1.902–4.075)		2.023(1.152–3.551)		2.102(0.964–4.584)	
**Radiotherapy**		0.664		**0.019**		0.108
Yes	Reference		Reference		Reference	
No	0.882(0.500–1.555)		5.988(1.350–26.560)		5.588(0.685–45.615)	

*SCNCC, small-cell neuroendocrine carcinoma of the cervix; SCNCO, small-cell neuroendocrine carcinoma of the ovary; SCNCE, small-cell neuroendocrine carcinoma of the endometrium all factors with Unknown data were removed in logistic regression model. Bold value means p < 0.05.*

**FIGURE 1:**
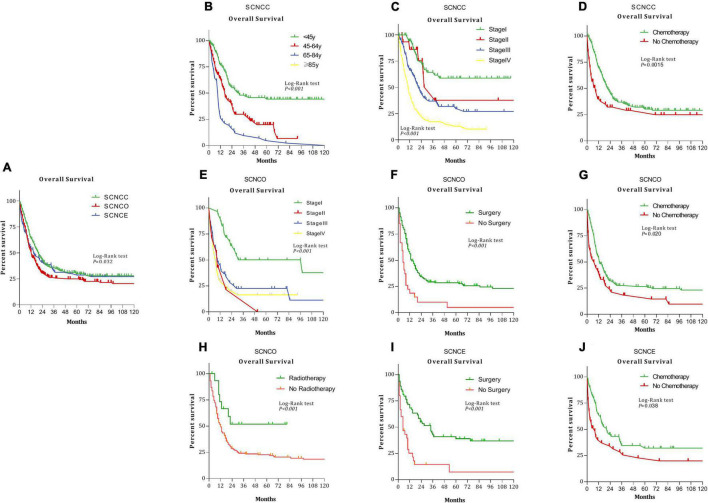
Kaplan–Meier analysis of overall survival among patients with small-cell neuroendocrine carcinoma of the gynecologic tract (SCNCGT) (*P* = 0.032) **(A)**. Small-cell neuroendocrine carcinoma of the cervix (SCNCC) and age (*P* < 0.001) **(B)**. SCNCC and stage (*P* < 0.001) **(C)**. SCNCC and chemotherapy (*P* = 0.0015) **(D)**. Small-cell neuroendocrine carcinoma of the ovary (SCNCO) and stage (*P* < 0.001) **(E)**. SCNCO and surgery (*P* = 0.0015) **(F)**. SCNCO and chemotherapy (*P* = 0.020) **(G)**. SCNCO and radiotherapy (*P* < 0.001) **(H)**. Small-cell neuroendocrine carcinoma of the endometrium (SCNCE) and surgery (*P* = 0.0015) **(I)**. SCNCE and chemotherapy (*P* = 0.038) **(J)**.

The median overall survival time was 13 months for gynecologic small-cell neuroendocrine carcinoma. Median survival times were as follows: 19 months (95% CI: 16.03–21.96) for cervical, 11 months (95% CI: 8.94–13.06) for ovarian, and 12 months (95% CI: 7.17–16.84) for endometrial small-cell neuroendocrine carcinoma (Figure 1A, *P* = 0.032).

In Kaplan–Meier analysis, overall survival was worse for patients with cervical small-cell neuroendocrine carcinoma with advanced age (Figure 1B, *P* < 0.001), those with stage III and IV diseases (Figure 1C, *P* < 0.001) and those who had not undergone chemotherapy (Figure 1D, *P* = 0.0015). Survival outcomes were worse for patients with ovarian small-cell neuroendocrine carcinoma diagnosed with stage II disease (Figure 1E, *P* < 0.001) and those who had not undergone surgery (Figure 1F, *P* = 0.0015), chemotherapy (Figure 1G, *P* = 0.020), or radiotherapy (Figure 1H, *P* = 0.0015). Furthermore, overall survival was worse for patients with endometrial small-cell neuroendocrine carcinoma who had not undergone surgery (Figure 1I, *P* = 0.0015) or chemotherapy (Figure 1J, *P* = 0.038).

## Discussion

### Summary of Main Results

Our study showed that cervical cases were the most common type of gynecologic small-cell neuroendocrine carcinoma, followed by ovarian and endometrial cases. Women with gynecologic small-cell neuroendocrine carcinoma had poor overall survival, and long-term survival was rarely reported, with the highest survival rate observed in cervical cases. Advanced age, stage III and IV cancer, and no chemotherapy treatment were potential predictors of poor prognosis of cervical small-cell neuroendocrine carcinoma, while stage III and IV cancer and lack of surgery, radiotherapy, or chemotherapy were predictors of poor prognosis of ovarian small-cell neuroendocrine carcinoma.

### Results in the Context of Published Literature

Previous studies have reported that the incidence of gynecologic small-cell neuroendocrine carcinoma is 2% (4, 5). However, our analysis of SEER data yielded an incidence of only 0.125%. Our data confirmed that women with gynecologic small-cell neuroendocrine carcinoma had poor overall survival. For all women with gynecologic small-cell neuroendocrine carcinoma, the median survival time after diagnosis was only 15 months. The highest survival rate was observed among patients with cervical small-cell neuroendocrine carcinoma, who exhibited a median survival time of 19 months. The median survival times for ovarian and endometrial small-cell neuroendocrine carcinoma were 11 and 12 months, respectively. The survival rates observed in our study are extremely low when compared with those for female genital tract cancer (3). The 5-year survival rates were 26.1, 22.0, and 5.9% for cervical, ovarian, and endometrial small-cell neuroendocrine carcinoma, respectively. The morbidity and survival rates observed in our study are lower than those previously reported (1). Owing to low morbidity, imperfect treatment planning, and poor patient prognosis, early diagnosis and appropriate treatment are critical for improving patient survival.

Cervical small-cell neuroendocrine carcinoma typically presents as more advanced and with poorer prognosis than cervical squamous cell carcinoma or adenocarcinoma, and its recurrence rates are higher than those for either (6). Here, the median survival time of patients with cervical small-cell neuroendocrine carcinoma was only 19 months. In a 2019 multi-center neuroendocrine cancer study, Ishikawa et al. (7) investigated the prognostic factors and best treatments for early cervical small-cell neuroendocrine carcinoma. Their multivariate analysis revealed that T3/T4, paraaortic lymph node metastasis, distant metastasis, and the number of chemotherapy cycles were independent prognostic factors for survival (8). In a meta-analysis of 1,901 patients, radiotherapy was also associated with poor survival, while chemotherapy was associated with prolonged survival, similar to the findings observed for patients with small-cell lung cancer (9). These findings suggest that the first-choice treatment for early-stage cervical small-cell neuroendocrine carcinoma should be surgery with adjuvant chemotherapy either before or after surgery, and that clinicians should remain cautious when considering radiotherapy. Moreover, it should be noted that patient age and clinical stage were risk factors in our study, thus indicating that advanced age, T3/T4, and adjuvant chemotherapy can significantly increase death risk.

Etoposide plus cisplatin and irinotecan plus cisplatin are currently the best chemotherapy regimens for cervical small-cell neuroendocrine carcinoma (10, 11). However, recent studies have identified potential targeted therapies, including PD-1, PI3K, and MEK inhibitors (12–15). Whether these and other molecular targeting strategies can benefit patients with cervical small-cell neuroendocrine carcinoma requires further research.

The largest case series included 42 cases of endometrial neuroendocrine carcinoma (15). This Japanese study reported that patients with stage III and IV simple small-cell neuroendocrine carcinomas exhibited significantly poorer prognosis than those with less advanced disease. Multivariate analysis indicated that the histological subtype and surgery were important prognostic factors. Thus, if radical surgery is possible, it should be the primary treatment for endometrial small-cell neuroendocrine carcinoma. Our findings indicated that the median survival time of patients with endometrial small-cell neuroendocrine carcinoma was 12 months, with a 5-year survival rate of 25.9%. Factors associated with prognosis include not only surgery but also adjuvant chemotherapy and radiotherapy. Sawada et al. (16) reported the rare case of a patient with late endometrial small-cell carcinoma with liver and brain metastases who survived for 12 years. The patient underwent pelvic tumor reduction surgery and metastasis resection, postoperative treatment with irinotecan hydrochloride combined with cisplatin chemotherapy, and adjuvant radiotherapy. Viau et al. (17) presented the case of a patient diagnosed with stage IV uterine intimal small-cell carcinoma who underwent surgery, chemotherapy (cisplatin/etoposide), and radiotherapy. Five years after completing the treatment, the patient remained disease-free. These reports suggest that treatments based on surgery, adjuvant radiotherapy, and chemotherapy can improve patient survival.

Ovarian small-cell neuroendocrine carcinoma had the lowest median survival time among all gynecologic small-cell neuroendocrine carcinomas at only 11 months. The currently recommended chemotherapy regimen is a multi-drug combination of platinum and etoposide for postoperative chemotherapy (18, 19). In a study of 469 women, Nasioudis et al. (18) observed 5-year overall survival rates of 48.6, 30.7, 18, and 12.3% in patients with stages I–IV, respectively. Their multivariate analysis revealed that an earlier stage of the disease and chemotherapy use were associated with lower mortality, despite no association with lymph node dissection. This provides an important basis for performing lymph node dissection during ovarian small-cell neuroendocrine carcinoma. In our study, there were only 217 cases of ovarian small-cell neuroendocrine carcinoma, which had a 5-year overall survival rate of 22.0%. Based on these findings, adding surgery and radiotherapy to chemotherapy treatment may increase patient survival.

Although the SEER database does not mention the diagnosis and treatment of recurrent gynecologic small-cell neuroendocrine carcinoma, even early cases are associated with a 30% chance of recurrence (20). While there are international guidelines for diagnosing and treating cervical cancers, no such guidelines exist for diagnosing and treating recurrent cervical small-cell neuroendocrine carcinoma (21). In fact, the National Comprehensive Cancer Network Guidelines for the Treatment of Cervical Cancer clearly exclude advanced neuroendocrine cancers (22). Many scholars currently advocate combined chemotherapy using topotecan, paclitaxel, and bevacizumab. However, this approach is not particularly effective, with a median survival time of only 10 months (23). Currently, bevacizumab is the preferred chemotherapy drug and has been used in phase II clinical trials (24).

### Strengths and Weaknesses

This study investigated the prevalence, risk factors, and survival outcomes of gynecologic small-cell neuroendocrine carcinoma using the largest publicly available cancer data set. However, there were certain limitations. The SEER database only provides information on whether and when (i.e., before or after surgery) radiotherapy and chemotherapy were performed, while the specific plan of treatment is not provided. Therefore, the clinical data were not detailed enough to determine whether specific regimens were associated with prognosis/survival. We also noticed that the degree of differentiation of the disease was very low in stages I–II and more concentrated in stages III–IV. Despite the grade not being a significant predictor in univariate and multivariate Cox risk regression analyses, there may be a certain correlation between the low survival rate of the disease and low degree of pathological differentiation. Lastly, given the rarity of gynecologic small-cell neuroendocrine carcinoma, the literature consists of only case reports and small case series.

### Implications for Practice and Future Research

There is currently no conventional guideline, unified treatment plan, or method for prognostic evaluation for gynecologic small-cell neuroendocrine carcinoma. Large-scale multi-center studies are required to elucidate the biological behavior of the disease and explore the most suitable treatment methods for improving patient prognosis. However, future studies should focus on exploring gene-targeted therapy, improving the preoperative diagnosis rate, and developing appropriate treatment guidelines.

## Conclusion

The survival times of endometrial and ovarian small-cell neuroendocrine carcinoma are relatively short, and long-term survival is rarely reported. Based on our findings, we believe that comprehensive and individualized treatment for gynecologic small-cell neuroendocrine carcinoma may prolong patient survival.

## Data Availability Statement

The datasets presented in this study can be found in online repositories. The names of the repository/repositories and accession number(s) can be found in the article/supplementary material.

## Ethics Statement

Ethical review and approval was not required for the study of human participants in accordance with the local legislation and institutional requirements.

## Author Contributions

LP collected clinical data and wrote the manuscript. SZW helped design and revise the manuscript. Both authors contributed to the article and approved the submitted version.

## Conflict of Interest

The authors declare that the research was conducted in the absence of any commercial or financial relationships that could be construed as a potential conflict of interest.

## Publisher’s Note

All claims expressed in this article are solely those of the authors and do not necessarily represent those of their affiliated organizations, or those of the publisher, the editors and the reviewers. Any product that may be evaluated in this article, or claim that may be made by its manufacturer, is not guaranteed or endorsed by the publisher.

## Footnotes

1
https://seer.cancer.gov/data/
